# Sinonasal-Related Orbital Infections in Children: A Clinical and Therapeutic Overview

**DOI:** 10.3390/jcm8010101

**Published:** 2019-01-16

**Authors:** Sara Torretta, Claudio Guastella, Paola Marchisio, Tal Marom, Samantha Bosis, Tullio Ibba, Lorenzo Drago, Lorenzo Pignataro

**Affiliations:** 1 Otolaryngological Unit, Fondazione IRCCS Ca’ Granda Ospedale Maggiore Policlinico, 20122 Milan, Italy; caludio.guastella@unimi.it (C.G.); paola.marchisio@unimi.it (P.M.); samantha.bosis@policlinico.mi.it (S.B.); tullio.ibba@policlinico.mi.it (T.I.); lorenzo.pignataro@unimi.it (L.P.); 2 Department of Clinical Sciences and Community Health, University of Milan, 20122 Milan, Italy; 3 Department of Physiopathology and Transplantation, University of Milan, 20122 Milan, Italy; 4 Department of Otolaryngology-Head and Neck Surgery, Samson Assuta Ashdod University Hospital, Faculty of Health Sciences, Ben Gurion University, 7747629 Ashdod, Israel; talmarom73@gmail.com; 5 Department of Clinical Microbiology, University of Milan, 20122 Milan, Italy; lorenzo.drago@unimi.it

**Keywords:** orbital cellulitis, children, rhinosinusitis, computed tomography

## Abstract

Sinonasal-related orbital infections (SROIs) are typically pediatric diseases that occur in 3–4% of children with acute rhinosinusitis. They are characterised by various clinical manifestations, such as peri-orbital and orbital cellulitis or orbital and sub-periosteal abscesses that may develop anteriorly or posteriorly to the orbital septum. Posterior septal complications are particularly dangerous, as they may lead to visual loss and life-threatening events, such as an intracranial abscess and cavernous sinus thrombosis. Given the possible risk of permanent visual loss due to optic neuritis or orbital nerve ischemia, SROIs are considered ophthalmic emergencies that need to be promptly recognised and treated in an urgent-care setting. The key to obtaining better clinical outcomes in children with SROIs is a multi-disciplinary assessment by pediatricians, otolaryngologists, ophthalmologists, radiologists, and in selected cases, neurosurgeons, neurologists, and infectious disease specialists. The aim of this paper is to provide an overview of the pathogenesis, clinical manifestations, diagnosis, and treatment of pediatric SROIs, and to make some practical recommendations for attending clinicians.

## 1. Introduction and Definitions

Sinonasal-related orbital infections (SROIs) are typically pediatric diseases that occur in about 3–4% of children with acute rhinosinusitis [1], and mainly affect children aged less than five years [2]. They are generally due to ethmoidal sinusitis spreading to the orbit, and are more frequent during winter.

**Figure 1:**
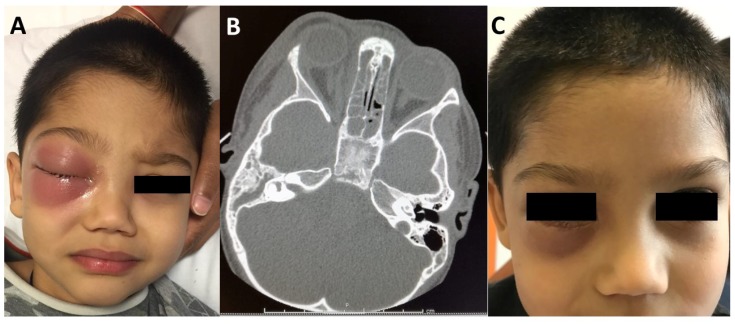
(**A**) A 3-year-old patient with a right sub-periosteal abscess extending to the lower lid and causing proptosis. (**B**) Computed tomography (CT) findings demonstrating bilateral ethmoiditis and sphenoidal sinusitis—the white arrow indicates the presence of right ethmoiditis, while the black arrow indicates a sub-periosteal abscess of the intra-orbital portion of the *lamina papyracea*. (**C**) The same patient 10 days after combined endoscopic surgery and external lower lid incision.

**Figure 2:**
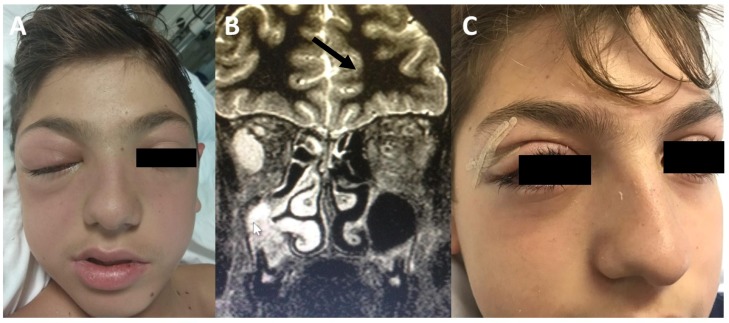
(**A**) A 13-year-old patient with acute maxillary sinusitis, an orbital abscess, and meningitis. (**B**) MR findings—the white arrow indicates the presence of right maxillary sinusitis, while the black arrow indicates an intraconal abscess located in the lateral portion of the orbit. (**C**) The same patient seven days after combined endoscopic surgery and an external upper lid incision.

SROIs are characterised by various clinical manifestations that may develop anteriorly or posteriorly to the orbital septum, a thin fibrous membrane extending from the orbital rims to the eyelids (the anterior boundary of the orbit) that acts as a barrier against the spread of external infections to the deep orbit. Chandler’s classification [3] distinguishes pre-septal complications, such as pre-septal cellulitis (POC, Chandler stage 1), which affects the eyelids and adnexa, without extending beyond the peri-orbit, from the more dangerous infections that develop posteriorly to the orbital septum, i.e., orbital cellulitis (OC, Chandler stage 2), sub-periosteal abscess (SPA, the collection of pus in the *lamina papyracea*, Chandler stage 3) (Figure 1), or orbital abscess (OA, Chandler stage 4) (Figure 2). Chandler stage 5 disease refers to cavernous sinus thrombosis (Table 1). Posterior septal complications are particularly dangerous, as they may lead to visual loss and life-threatening events, such as an intracranial abscess and cavernous sinus thrombosis. SPA accounts for 9–28% of all SROIs [4,5], and is generally secondary to ethmoiditis; it is more frequently located in the medial portion of the orbit [6], but other less frequent locations include superior and superomedial SPA generally due to frontal sinusitis [6]. Some authors have suggested that osteitis of the *lamina papyracea* occurring during acute ethmoiditis may play an etiological role in the development of SPA [7], as it is detected in more than 55% of cases.

However, it has been suggested that SROIs may be clinically better defined by the presence or absence of any abscess, and its location within the orbit or in the sub-periosteal plane, rather than by discriminating its pre- and post-septal extension [8]. 

The clinical presentation of SROIs includes swelling and redness around the eye, and possibly a temperature and impaired general condition, which are generally more frequent in the case of orbital involvement. The clinical signs suggesting post-septal complications due to increased intra-orbital pressure are proptosis, chemosis, ophthalmoplegia, diplopia, impaired visual acuity, or impaired red-green visual perception [9]. Although proptosis and ophthalmoplegia are strong predictors of advanced disease [10], computed tomography (CT) findings cannot differentiate OC from OA, and may be absent in some patients with intra-orbital involvement [10]. It has been reported that proptosis and ophthalmoplegia may be absent in up to 50% of patients in Chandler stage ≥3 [11], and so some authors have suggested that, in addition to a clinical examination, consideration should be given to other predictors, such as a high neutrophil count, older age, and a body temperature of >39 °C [10,12].

SROIs are ophthalmic emergencies that need to be promptly recognised and treated in an urgent-care setting because of the possible risk of permanent visual loss due to optic neuritis or orbital nerve ischemia following orbital compression or stretching secondary to sub-periosteal or intra-orbital collections or orbital vein thrombosis. Life-threatening sequelae, such as cavernous sinus thrombosis, meningitis, and epidural or sub-dural abscess, may also occur as a result of intracranial spreading [13,14,15].

The aim of this paper is to provide an overview of the pathogenesis, diagnosis, and treatment of pediatric SROIs, and make some practical recommendations for attending clinicians.

## 2. Etiopathogenesis and Differential Diagnosis

Unlike adults (in whom SROIs are less common and are generally due to frontal sinonasal disease) [13], children with SROIs are mainly affected by acute ethmoiditis, because the development of the frontal (and maxillary) sinuses is completed by the age of 10–12 years, whereas the ethmoid labyrinth is generally well developed and pneumatised in newborns [13]. It must also be remembered that the *lamina papyracea* (the lateral wall of the ethmoid sinus and its boundary with the adjacent orbital cavity) is generally thinner and more porous in children, and that venous outflow from the ethmoid to the intracranial district throughout the orbit can allow the passage of hematogenous infections in both an antero- and retrograde direction [2].

Although complicated sinonasal disease is the most frequent etiological factor in children with orbital or peri-orbital cellulitis (its reported incidence ranged from 60% to 91% of cases) [16,17], orbital swelling alone is non-specific and not pathognomonic for complicated sinusitis, and so physicians should also consider other conditions that may evolve into even severe disease. These include dacryocystitis or dacryoadenitis, dental abscesses, insect bites or stings, maxillofacial and orbital traumas, foreign bodies, neoplasms [12,13], allergic conjunctivitis or allergic reactions, acute sickle cell orbitopathy (i.e., orbital infarctions or sub-periosteal hematomas in patients with sickle cell disease), and idiopathic orbital inflammatory disease [18,19,20,21]. SROIs should also be differentiated from other infrequent conditions possibly causing peri-orbital swelling, such as orbital pseudo-tumour, orbital myositis, Wegener’s granulomatosis, sarcoidosis, leukemia, Burkitt’s lymphoma, histiocytosis, and dysthyroid orbitopathy [2].

SROIs are generally due to the bacteria involved in acute rhinosinusitis: the species most frequently isolated from purulent secretions are *Haemophilus influenzae* type B, *Streptococcus pneumoniae*, *Staphylococcus aureus*, *Staphylococcus epidermidis*, and *Streptococcus pyogenes* [22]. According to some studies, *Streptococcus* group A and *Streptococcus anginosus* are occasionally isolated from pus collected from SPAs and seem to predispose patients to intracranial complications [23]. Other authors have reported a high prevalence of *Staphylococcus aureus* in samples collected from patients with SROIs, and have highlighted the emerging etiological role of methicillin-resistant strains [24].

Furthermore, even after the introduction of *Haemophilus influenzae* type B vaccination, non-typeable *Haemophilus influenzae* has been documented in about 13% of cultures of fluid collected after the surgical drainage of an orbital abscess, and is still a major causal agent [15]. 

## 3. Diagnosis

A multi-disciplinary clinical assessment should evaluate a patient’s general appearance (in order to detect fever and any signs of possible systemic toxicity or neurological involvement, such as seizures, focal neurological defects, vomiting, or altered mental status) and local conditions, taking particular care to differentiate pre- and post-septal infections (Figure 1 and Figure 2). Visual acuity and eye movements should be assessed on a daily basis. Although there is no consensus concerning the ideal frequency of visual examination (with suggestions ranging from every two hours to twice a day) [25], at least twice a day is recommended [14]. Fundoscopy should also be considered in the case of suspected optic nerve impairment. Children with pre-septal involvement are generally systemically well, but signs of systemic involvement (including fever) may occur more frequently if the infection spreads beyond the orbital septum [26].

A local examination generally reveals unilateral eyelid and peri-orbital swelling, tenderness, erythema, and warmth. The clinical signs suggesting possible intra-orbital involvement are an inability to open the eye sufficiently to allow examination, proptosis, ophthalmoplegia, and diplopia, impaired vision (reduced acuity or the loss of perception of the colour red), asymmetrical pupillary reactivity, chemosis, or injection of the conjunctiva or sclera [26]. An otolaryngological examination using nasal fibre endoscopy is useful for exploring the nasal cavities, the middle and superior meatus and osteomeatal complex, and the sphenoethmoidal recess, in order to detect any sign of sinus disease (including mucosal edema and purulent secretion) and guide the collection of samples for microbiological analysis.

Children with uncomplicated POC do not need to undergo routine baseline imaging [27], but if available and provided that it does not delay any further treatment, urgent magnetic resonance imaging (MRI) with gadolinium enhancement is the method of choice for localising and diagnosing an abscess in the case of suspected intra-orbital involvement (or if the impossibility of inspecting the eye during a bedside examination cannot rule it out), clinical deterioration, no response to intravenous antibiotic treatment after >48 h, or suspected intracranial extension. The alternative is contrast-enhanced computed tomography (CT) of the orbit, the maxillo-facial unit, and the brain [26,28]. The diagnostic accuracy of CT in detecting OC, OA, or SPA ranges from 91% to 100% [29,30], and its pre-surgical use is essential to plan the treatment of the primary sinonasal focus and guide the surgical drainage of any intra-orbital or sub-periosteal collection. It can also aid surgeons in identifying the level of the cribriform plate, and any abnormal bony and vascular structures. 

In the case of a neurological deficit, persistent fever despite adequate treatment, or positive brain CT findings, an urgent MRI scan of the brain and orbits should be carried out provided that it does not delay further treatment.

Laboratory tests, including assessment of white blood cell counts and serum C-reactive protein levels may be useful, as inflammatory markers are generally higher in children with SROIs than in those with peri-orbital cellulitis unrelated to complicated rhinosinusitis; it has also been reported that the presence of fever, a white blood cell count of >11,100 per microlitre, and proptosis are independent predictors of SPA or OA [31].

In the case of suspected sepsis or meningitis, further investigations, such as blood cultures and a lumbar puncture, should be considered [32].

## 4. Treatment

Hospital admission should be proposed even in initial disease stages, because given the possibility of the infection spreading to the intra-orbital or intracranial compartment, this would allow close in-house clinical monitoring. There is no agreement concerning the ideal treatment of SROI, but current evidence suggests that parenteral medical treatment should be considered first and immediately started in the case of POC [14]. Conservative treatment with close clinical monitoring should also be considered in children with OC [33].

The initial medical treatment consists of intravenous antibiotics that cover the most widely involved pathogens and provide adequate penetration of the central nervous system in order to decrease the risk of intracranial diffusion. These schedules include clindamycin plus third-generation cephalosporin, vancomycin with or without meropenem, ampicillin-sulbactam, and third-generation cephalosporin plus metronidazole [13,14,34,35,36].

Topical intranasal medication with decongestants is essential to improve the patency of the osteomeatal complex and facilitate sinonasal drainage; nasal irrigations with saline or (better) hypertonic solutions should be considered as complementary treatments.

In the case of multiple foci of bacterial infections, children aged >9 years, or those with known immunodeficiency, surgery should be considered if there is no improvement >48 h after parenteral antibiotic treatment [16,37]. SPA and OC (with or without intracranial complications) are considered emergencies and were traditionally managed by means of immediate surgical drainage; however, the therapeutic management of SPA has recently been widely debated and there is no consensus concerning the need for or timing of surgery. 

The published success rates of the different means of treating SPA range from 26% to 94.5% [38,39,40,41]. A conservative medical approach with close observation is proposed for patients without impaired visual acuity or increased intra-ocular pressure, with the option of surgery in the case of worsening ophthalmological findings or a lack of improvement after 48 h [13]. Some evidence suggests that medical treatment is more effective in children aged <9 years than in older children [41,42]. It has also been reported that SPA volume as revealed by CT is a significant prognostic factor that can be considered when selecting patients for surgery, but the cut-off values above which surgery is required very widely, from 0.48 mL to 3.8 mL [18,37,43]. Abscess width has been investigated as a possible criterion for surgery, but the results are conflicting—some authors have found that abscesses of <10 mm can be successfully managed by means of medical treatment alone [5], whereas others advocate immediate surgery in the case of abscesses of ≥4 mm [40]. However, most authors [39,43,44] strongly recommend the early surgical treatment of any SPA of ≥500 mm^3^ (~0.5 mL), because although it has been reported that these can be effectively managed medically, it requires longer hospitalisation and the administration of antibiotics [44]. Different cut-off values of SPA volume for surgical indication have been proposed, but the results cannot be compared because of the different criteria used to quantify the exact volume.

In terms of surgical approaches, endoscopic sinus surgery (ESS) has proved to be effective in treating SPA and OA, with the surgical goals of draining the abscess, restoring intra-orbital pressure and the patency and drainage of the sinonasal complex, and collecting samples for culture. The surgical steps include uncinectomy, middle meatal antrostomy, ethmoidectomy, and penetration of the *lamina papyracea*. However, although this procedure seems to be appropriate in the case of medial locations (including those with a posterior extension) [39,43,45], it is generally believed that a superomedial location can be better managed by combining ESS with an external superior orbital rim incision [40,43,45]. Superiorly located SPAs generally require an external approach [43], but a combined endonasal endoscopic and external approach should be considered in the case of the most laterally located superior SPAs [43].

## 5. Conclusions and Practical Recommendations

**Figure 3:**
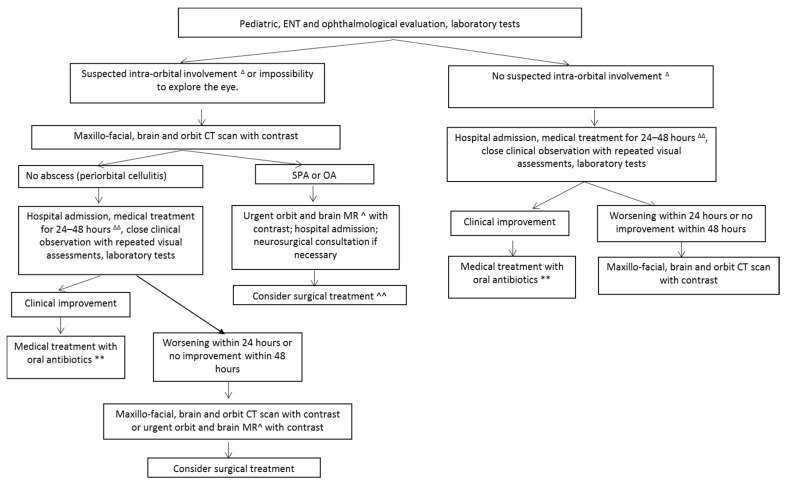
Diagnostic and therapeutic strategies in patients with sinonasal-related orbital infections. ENT: ear, nose and throat; SPA: subperiosteal abscess; OA: orbital abscess; CT: computed tomography; MR: magnetic resonance. ^∆^ Lid swelling and redness with proptosis, eyeball displacement, impaired ocular motility, visual loss, chemosis. ^∆∆^ Topical intranasal medication with decongestants, parenteral antibiotics (ampicillin-sulbactam (100 mg/kg/die in three doses) or cefotaxime (100 mg/kg/die in three doses) or cephtriaxone (100 mg/kg/die in one dose). ** Amoxicillin plus clavulanic acid (90 mg/kg/die in three doses) for two weeks. ^ Provided that it does not delay surgery. ^^ In the case of a small SPA without impaired visual acuity or increased intra-ocular pressure, surgery can be delayed until after the failure of medical treatment.

The key to obtaining better clinical outcomes in children with SROIs is a multidisciplinary assessment by pediatricians, otolaryngologists, ophthalmologists, radiologists, and in selected cases, neurosurgeons, neurologists, and infectious disease specialists (Figure 3). Hospital admission and close clinical and ophthalmological observation, particularly aimed at ensuring the prompt detection of any sign suggesting intra-orbital involvement, is essential. Imaging studies should be considered in the case of suspected intra-orbital or intracranial extension, the impossibility of inspecting the eye, clinical deterioration, or a clinical non-response to 24–48 h of antibiotic treatment.

Intravenous antibiotic treatment should be started immediately, and can be effective as first-line therapy in patients with POC and OC. 

Surgical treatment by means of ESS or an external approach, or both, depending on location, should be used in patients with SPA, OC, or intracranial complications in an emergency setting. It should also be considered if no improvement is observed 24–48 h after parenteral antibiotic treatment in the case of multiple bacterial infections, and in children aged >9 years or those with known immunodeficiency [16,37].

In patients with a small SPA without impaired visual acuity or increased intra-ocular pressure, surgery can be considered as a second step in the case of worsening ophthalmological findings or no improvement after 48 h.

## Figures and Tables

**Table 1:** Chandler’s classification of sinonasal-related orbital infections [3]. Note: * = cellulitis affecting the eyelids and adnexa without extending beyond the peri-orbit; ** = collection of pus between the periorbita and the orbital wall.

Stage	Description
1	Pre-septal cellulitis *
2	Orbital cellulitis
3	Sub-periosteal orbital abscess **
4	Orbital abscess
5	Cavernous sinus thrombosis

## References

[1] Giusan A.O., Kubanova A.A., Uzdenova R.K. (2010). Rhinosinusogenic orbital complications: The prevalence and principles of treatment. Vestn. Otorinolaringol..

[2] Hauser A., Fogarasi S. (2010). Periorbital and orbital cellulitis. Pediatr. Rev..

[3] Chandler J.R., Langenbrunner D.J., Stevens E.R. (1970). The pathogenesis of orbital complications in acute sinusitis. Laryngoscope.

[4] Sobol S.E., Marchand J., Tewfik T.L., Manoukian J.J., Schloss M.D. (2002). Orbital complications of sinusitis in children. J. Otolaryngol..

[5] Ryan J.T., Preciado D.A., Bauman N., Pena M., Bose S., Zalzal G.H., Choi S. (2009). Management of pediatric orbital cellulitis in patients with radiographic findings of subperiosteal abscess. Otolaryngol. Head Neck Surg..

[6] Migirov L., Yakirevitch A., Bedrin L., Wolf M. (2009). Endoscopic sinus surgery for medial orbital subperiosteal abscess in children. J. Otolaryngol. Head Neck Surg..

[7] Eviatar E., Sandbank J., Kleid S., Gavriel H. (2014). The role of osteitis of the lamina papyracea in the formation of subperiosteal orbital abscess in young children. Int. J. Pediatr. Otorhinolaryngol..

[8] Velasco E., Cruz A.A., Demarco R.C., dos Santos A.C., Anselmo-Lima W.T., Marquezini R.M. (2007). Orbital complications of acute rhinosinusitis: A new classification. Braz. J. Otorhinolaryngol..

[9] Fokkens W.J., Lund V.J., Mullol J., Bachert C., Alobid I., Baroody F., Cohen N., Cervin A., Douglas R., Gevaert P. (2012). EPOS 2012, European position paper on rhinosinusitis and nasal polyps 2012. A summary for otorhinolaryngologists. Rhinology.

[10] Jabarin B., Eviatar E., Israel O., Marom T., Gavriel H. (2018). Indicators for imaging in periorbital cellulitis secondary to rhinosinusitis. Eur. Arch. Otorhinolaryngol..

[11] Rudloe T.F., Harper M.B., Prabhu S.P., Rahbar R., Vanderveen D., Kimia A.A. (2010). Acute periorbital infections: Who needs emergent imaging?. Pediatrics.

[12] Vu B.L., Dick P.T., Levin A.V., Pirie J. (2003). Development of a clinical severity score for preseptal cellulitis in children. Pediatr. Emerg. Care.

[13] Bedwell J., Bauman N.M. (2011). Management of pediatric orbital cellulitis and abscess. Curr. Opin. Otolaryngol. Head Neck Surg..

[14] Kinis V., Ozbay M., Bakir S., Yorgancilar E., Gun R., Akdag M., Sahin M., Topcu I. (2013). Management of orbital complications of sinusitis in pediatric patients. J. Craniofac. Surg..

[15] Sharma A., Liu E.S., Le T.D., Adatia F.A., Buncic J.R., Blaser S., Richardson S. (2015). Pediatric orbital cellulitis in the Haemophilus influenzae vaccine era. J. AAPOS.

[16] Vairaktaris E., Moschos M.M., Vassiliou S., Baltatzis S., Kalimeras E., Avgoustidis D., Pappas Z., Moschos M.N. (2009). Orbital cellulitis, orbital subperiosteal and intraorbital abscess: Report of three cases and review of the literature. J. Craniomaxillofac. Surg..

[17] Babar T.F., Zama M., Khan M.N., Khan M.D. (2009). Risk factors of preseptal and orbital cellulitis. J. Coll. Physicians Surg. Pak..

[18] Le T.D., Liu E.S., Adatia F.A., Buncic J.R., Blaser S. (2014). The effect of adding orbital computed tomography findings to the Chandler criteria for classifying pediatric orbital cellulitis in predicting which patients will require surgical intervention. J. AAPOS.

[19] Chaudhry I.A., Al-Rashed W., Arat Y.O. (2012). The hot orbit: Orbital cellulitis. Middle East Afr. J. Ophthalmol..

[20] Moubayed S.P., Vu T.T., Quach C., Daniel S.J. (2011). Periorbital cellulitis in the pediatric population: Clinical features and management of 117 cases. J. Otolaryngol. Head Neck Surg..

[21] Galindo-Ferreiro A., Alkatan H.M., ElKhamary S.M., AlDosairi S., Cruz A.A.V. (2017). Recurrent Orbital Inflammation Mimicking Orbital Cellulitis Associated with Orbitopalpebral Venous Lymphatic Malformation. Ophthalmic Plast. Reconstr. Surg..

[22] Botting A.M., McIntosh D., Mahadevan M. (2008). Paediatric pre- and post-septal peri-orbital infections are different diseases. A retrospective review of 262 cases. Int J Pediatr Otorhinolaryngol.

[23] Constatinides F., Luzzati R., Tognetto D., Bazzocchi G., Biasotto M., Tirelli G.C. (2012). Rapidly progressing subperiostal abscess: An unexpected complication of a group A streptococcal pharyngitis in a healthy young patient. Head Face Med..

[24] Pandian D.G., Babu R.K., Chaitra A., Anjali A., Rao V.A., Srinivasan R. (2011). Nine years’ review on preseptal and orbital cellulitis and emergence of community-acquired methicillin-resistent Staphylococcus aureus in a tertiary hospital in India. Indian J. Ophthalmol..

[25] Rubin S.E., Rubin L.G., Zito J., Goldstein M.N., Eng C. (1989). Medical management of orbital subperiosteal abscess in children. J. Pediatr. Ophthalmol. Strab..

[26] Adamson J., Waterfield T. (2018). Fifteen-minute consultation: Preseptal and orbital cellulitis. Arch. Dis. Child. Educ. Pract. Ed..

[27] Ho C.F., Huang Y.C., Wang C.J., Chiu C.H., Lin T.Y. (2007). Clinical analysis of computed tomography-staged orbital cellulitis in children. J. Microbiol. Immunol. Infect..

[28] Mathew A.V., Craig E., Al-Mahmoud R., Batty R., Raghavan A., Mordekar S.R., Chan J., Connolly D.J. (2014). Paediatric post-septal and pre-septal cellulitis: 10 years’ experience at a tertiary-level children’s hospital. Br. J. Radiol..

[29] Younis R.T., Lazar R.H., Bustillo A., Anand V.K. (2002). Orbital infection as a complication of sinusitis: Are diagnostic and treatment trends changing?. Ear Nose Throat J..

[30] Teinzer F., Stammberger H., Tomazic P.V. (2015). Transnasal endoscopic treatment of orbital complications of acute sinusitis: The Graz concept. Ann. Otol. Rhinol. Laryngol..

[31] Welkoborsky H.J., Graß S., Deichmüller C., Bertram O., Hinni M.L. (2015). Orbital complications in children: Differential diagnosis of a challenging disease. Eur. Arch. Otorhinolaryngol..

[32] NICE Sepsis: Recognition, Diagnosis and Early Management, NICE. https://www.nice.org.uk/guidance/ng51/resources.

[33] Wan Y., Shi G., Wang H. (2016). Treatment of Orbital Complications Following Acute Rhinosinusitis in Children. Balkan Med. J..

[34] Hongguang P., Lan L., Zebin W., Guowei C. (2016). Pediatric nasal orbital cellulitis in Shenzhen (South China): Etiology, management, and outcomes. Int J. Pediatr. Otorhinolaryngol..

[35] Coudert A., Ayari-Khalfallah S., Suy P., Truy E. (2018). Microbiology and antibiotic therapy of subperiosteal orbital abscess in children with acute ethmoiditis. Int. J. Pediatr. Otorhinolaryngol..

[36] Lin C.Y., Chiu N.C., Lee K.S., Huang F.Y., Hsu C.H. (2013). Neonatal orbital abscess. Pediatr. Int..

[37] Todman M.S., Enzer Y.R. (2011). Medical management versus surgical intervention of pediatric orbital cellulitis: The importance of subperiosteal abscess volume as a new criterion. Ophthalmic Plast. Reconstr. Surg..

[38] Souliere C.R., Antoine G.A., Martin M.P., Blumberg A.I., Isaacson G. (1990). Selective non-surgical management of subperiosteal abscess of the orbit: Computerized tomography and clinical course as indication for surgical drainage. Int. J. Pediatr. Otorhinolaryngol..

[39] Rahbar R., Robson C.D., Petersen R.A., DiCanzio J., Rosbe K.W., McGill T.J., Healy G.B. (2001). Management of orbital subperiosteal abscess in children. Arch. Otolaryngol. Head Neck Surg..

[40] Oxford L.E., McClay J. (2006). Medical and surgical management of subperiosteal orbital abscess secondary to acute sinusitis in children. Int. J. Pediatr. Otorhinolaryngol..

[41] Garcia G.H., Harris G.J. (2000). Criteria for nonsurgical management of subperiosteal abscess of the orbit: Analysis of outcomes. Ophthalmology.

[42] Liao J.C., Harris G.J. (2015). Subperiosteal abscess of the orbit: Evolving pathogens and the therapeutic protocol. Ophthalmology.

[43] Gavriel H., Yeheskeli E., Aviram E., Yehoshua L., Eviatar E. (2011). Dimension of subperiosteal orbital abscess as an indication for surgical management in children. Otolaryngol. Head Neck Surg..

[44] Nation J., Lopez A., Grover N., Carvalho D., Vinocur D., Jiang W. (2017). Management of Large-Volume Subperiosteal Abscesses of the Orbit: Medical vs Surgical Outcomes. Otolaryngol. Head Neck Surg..

[45] Froehlich P., Pransky S.M., Fontaine P., Stearns G., Morgon A. (1997). Minimal endoscopic approach to subperiosteal orbital abscess. Arch. Otolaryngol. Head Neck Surg..

